# Value of NT-proBNP and Galectin-3 as Biomarkers in the Follow-Up of Asymptomatic Elderly Patients with Severe Aortic Stenosis

**DOI:** 10.3390/jcm12082987

**Published:** 2023-04-20

**Authors:** Mónica Ramos, Maribel Quezada-Feijoó, Rocío Ayala, Ascensión Manzano, Francisco Javier Gómez-Pavón, Javier Jaramillo, Cristina Herrera, Mariola López Vazquez de la Torre, Rocío Toro

**Affiliations:** 1 Cardiology Department, Hospital Central de la Cruz Roja, C/Reina Victoria, 24, 28003 Madrid, Spain; maribelquezada2000@gmail.com (M.Q.-F.);; 2 Medicine School, Universidad Alfonso X El Sabio, Avda. De la Universidad, 1, Villanueva de la Cañada, 28691 Madrid, Spain; javiergomezpav@gmail.com (F.J.G.-P.);; 3 Geriatric Department, Hospital Central de la Cruz Roja, C/Reina Victoria, 24, 28003 Madrid, Spain; 4 Biochemistry Laboratory, Hospital Central de la Cruz Roja, C/Reina Victoria, 24, 28003 Madrid, Spain; 5 Departmental Section of Biochemistry and Molecular Biology, Faculty of Pharmacy, Universidad Complutense de Madrid, 28040 Madrid, Spain; 6 Research Unit, Biomedical Research and Innovation Institute of Cadiz (INiBICA), Puerta del Mar University Hospital, Av/Ana de Viya 21, 11009 Cádiz, Spain; rociotorogreen@gmail.com; 7 Medicine Department, School of Medicine, Cádiz University, Edificio Andrés Segovia 30 Floor, C/Dr. Marañón S/N, 21001 Cádiz, Spain

**Keywords:** aortic stenosis, older patients, congestive heart failure, Galectin-3, NT-proBNP

## Abstract

Recognizing symptoms in elderly patients with severe aortic stenosis (AS) can be a challenge. Serum biomarkers such as Galectin-3 or N-terminal prohormone B-type natriuretic peptide (NT-proBNP) are involved in remodeling and heart failure (HF) development and could support the diagnosis of AS. We set out to test the usefulness of NT-proBNP and Galectin-3 in predicting events in this population. We designed a prospective observational case–control study, including 50 asymptomatic patients older than 70 years, diagnosed with severe degenerative AS, and 50 control individuals. The NT-proBNP and Galectin-3 levels were measured. A follow-up was carried out at 12 months to determine the occurrence of hospital admission for HF, all-cause mortality or the appearance of symptoms. The patients with severe AS had higher Galectin-3 and NT-proBNP concentrations. The area under the receiver operating characteristic curve of the NT-proBNP was 0.812 (95% CI, 0.646–0.832), and that of the Galectin-3 was 0.633 (95% CI, 0.711–0.913). NT-proBNP was a good predictor of events [HR 3.45 (95% CI 1.32–9.03), *p* = 0.011]. A Kaplan–Meier analysis showed that the probability of freedom from events was significant in patients who exhibited a combination of higher NT-proBNP and Galectin-3 levels (log-rank *p* = 0.032). Therefore, NT-proBNP was the most reliable predictor of events in asymptomatic patients with severe AS. A combination of NT-proBNP and Galectin-3 levels may be vital in the clinical follow-up of these patients and in the decision-making process.

## 1. Introduction

Aortic stenosis (AS) is the most frequent valve pathology in clinical practice [1]. A degenerative etiology is currently the most prevalent cause of AS; furthermore, due to the aging of the population, there has been an increase in the number of diagnosed cases. Degenerative AS is a chronic and progressive entity with a long latency period during which the patient is asymptomatic. The duration of this asymptomatic period varies widely among patients, but, once symptoms appear, the prognosis worsens, and the 5-year survival rate is 15–50% [2,3].

Currently, two invasive treatment options are available for patients with severe AS, aortic valve replacement surgery (AVR) and transcatheter aortic valve implantation (TAVI); the former is more appropriate for patients older than 75 years and those with intermediate and high surgical risk, as long as there is no risk of failure [4].

Diagnosing AS in the elderly is challenging. First, recognizing symptoms in older patients can be difficult, due to the presence of comorbidities that negatively influence physical activity or because the patients unconsciously limit themselves physically in such a way that they do not report any symptoms. In addition, the collection of medical history data could be even more complicated if this aging population presents with cognitive impairment such as dementia or delirium [5,6]; therefore, by the time they begin to notice any limitations, the AS is already seriously advanced [7]. Second, performing diagnostic tests such as stress tests in this population is complicated, since many patients have difficulty with the treadmill, are incapable of performing the tests or are contraindicated by patient comorbidities. In recent years, the use of circulating biomarkers such as N-terminal prohormone B-type natriuretic peptide (NT-proBNP) to predict symptom-free survival has been proposed. NT-proBNP is related to hemodynamic AS severity and may allow the early identification of patients who will have a worse evolution and prognosis and may benefit from early invasive treatment. Thus, NT-proBNP serves as a prognostic tool and determines the best time for surgery [4,8,9]. However, NT-proBNP levels do not reflect ongoing chronic inflammation and appear to have lower specificity in older patients [10].

Galectin-3 belongs to the β-galactoside-binding protein family and serves important functions in numerous biological activities, including cell growth, apoptosis, differentiation, inflammation and fibrosis [11]. Recent studies support the use of this emerging biological marker for the prediction of survival in asymptomatic AS cohorts due to its role in remodeling and the development of congestive heart failure (CHF); however, to our knowledge, no specific studies have been carried out assessing the efficacy of Galectin-3 in the elderly population [12,13,14].

The identification of high-risk populations is critical to avoid readmission and high-rate mortality and thus achieve effective management and use of resources. However, indications for invasive medical treatment among frail elderly patients with severe AS are limited because of the lack of symptoms, difficulties in performing diagnostic tests or the condition itself, which can cause delayed timing. In the current study, we aimed to test the usefulness of NT-proBNP and Galectin-3 in an older population with asymptomatic severe AS.

## 2. Materials and Methods

### 2.1. Population Study

This is a prospective, observational, case–control study. Two cohorts were recruited: (i) patients older than 70 years diagnosed with asymptomatic severe degenerative AS (n = 50) and (ii) healthy controls who were matched by age and sex. The patients were recruited from cardiology outpatient clinics. Patients with severe concomitant valvulopathies, patients under 70 years of age, and patients with severe AS who were symptomatic or pending intervention were excluded. The control group consisted of individuals who showed a normal clinical examination and did not present with any chronic heart condition except high blood pressure (HBP). The case recruitment was performed from August 2017 to July 2018, and the control cohort was included from December 2020 to May 2021.

The study protocol was approved by the Ethics Committee of Alfonso X el Sabio University/UAX and met the ethical criteria of the Declaration of Helsinki. Signed informed consent was obtained from all patients.

### 2.2. Clinical Data

Biodemographic data were collected, including age, sex, weight, height, body mass index (BMI) and body surface area (BSA), using the DuBois and DuBois formula. The clinical variables analyzed included unhealthy habits and cardiovascular risk factors such as HBP, diabetes mellitus (DM) or dyslipidemia (DLP). The presence of atrial fibrillation (AF), ischemic heart disease, and comorbidities included in the Charlson index were also collected [15]. A physical examination and a 12-lead electrocardiogram were performed. Dyspnea severity was classified according to the New York Heart Association (NYHA) functional classification. The patients were considered asymptomatic in the absence of dyspnea, angina and syncope. The degree of dependency was measured by the Barthel scale [16].

### 2.3. Echocardiography Information

All the patients underwent comprehensive Doppler echocardiography on study recruitment with a Phillips Affinity-70C echocardiograph with an S5-1 probe by an experienced echocardiographer. The severity of aortic valve disease was calculated following the recommendations of the European Society of Cardiology by evaluating the maximum velocity (Vmax), the mean pressure gradient (MPG) and the aortic valve area (AVA), calculated using the continuity equation and the dimensionless index (DI) or integral relation, which represents the ratio of the left ventricular outflow tract (LVOT) time-velocity integral to that of the aortic valve jet [4]. Severe AS was considered when the peak velocity was greater than 4 m/s, the MPG was greater than 40 mmHg, and the DI was less than 0.25, or when the AVA was less than 1 cm and the indexed AVA was less than 0.6 cm/m^2^ to also include severe AS with a low gradient [4,17].

Two-dimensional measurements of the left ventricular (LV) diameter and wall thickness and the left atrial volume were also performed. The left ventricular ejection fraction (LVEF) was calculated using the four-chamber and two-chamber Simpson biplane methods and was considered preserved above 50% and reduced when it was ≤50% [18].

The LV diastolic function and filling pressures were assessed according to the recommendations of the European Association of Cardiovascular Imaging [19]. The global longitudinal strain (GLS) was determined by 2D speckle tracking from apical 2, 3 and 4 chamber views according to the American Society of Echocardiography 17 segment LV model. In each of the apical views, three sampling points were manually placed at the endocardial edge of the septal and lateral mitral annulus and at the apex. The region of interest was automatically generated and manually edited, with the aim of obtaining optimal tracing of the endocardial and epicardial borders [20]. Representative cardiac cycles were chosen to determine longitudinal strain, with the goal of achieving the best myocardial tracking and the most visually satisfying strain curves. The aortic valve closure was visually identified from the apical long-axis view. The GLS was calculated as the average of each of the regional strains. The available automated function imaging software package was used for the analysis. The software only allowed the calculation of the GLS if the follow-up was adequate in at least five of six segments in each apical view. If more than three segments were not adequately followed up, the GLS analysis was not performed.

The size and function of the right ventricle and the estimation of systolic pulmonary arterial pressure (sPAP) were evaluated following the guidelines of the European Society of Cardiology [21].

### 2.4. Biochemical Analysis

All the blood samples were taken from a peripheral vein and processed under identical conditions at 08:00 h after 12 h of fasting and within 48 h of echocardiography. The Galectin-3 samples and NT-proBNP were analyzed at the laboratory using a fluorescent enzyme-linked immunoassay on the VIDAS analyzer (bioMérieux, Craponne, France).

The Galectin-3 level was classified into three groups according to the cutoff previously proposed for risk stratification in established HF. High risk was considered when it was >25.9 ng/mL, moderate risk > 17.8–25.9 ng/mL and low risk ≤ 17.8 ng/mL [22]. We also classified the Galectin-3 levels according to the cutoff point obtained in the study, as with the NT-proBNP.

The estimated glomerular filtration rate (eGFR) was calculated by the Chronic Kidney Disease Epidemiology Collaboration equation. Renal function was considered impaired when the eGFR < 60 mL/min/1.73 m^2^ [23].

### 2.5. Study Population Follow-Up

The recruited patients were followed up at six months and one year in cardiology clinics or by telephone when the patient was unable to attend. For evaluating the results, the endpoint of the study was defined as the occurrence of death from any cause, admission for CHF or the appearance of symptoms (dyspnea, angina or syncope) and a change in treatment (referral for TAVI or AVR). Likewise, the different causes of mortality were collected.

### 2.6. Statistical Analysis

Stata 15.1 software (www.stata.com accessed on 18 October 2022) was used for the statistical analysis. For the description of the data, the frequency and percentages are used for the qualitative variables, and the means (standard deviation), median (interquartile interval) and minimum/maximum are used for the quantitative variables. Comparisons between the quantitative variables were made using the independent samples Student’s t-test for the variables with a normal distribution according to the Kolmogorov–Smirnov test. For the variables that did not conform to normality, the Mann–Whitney U test was used. The qualitative variables are shown as total numbers and percentages. The comparisons between the qualitative variables were made using Pearson’s χ2 test with Yates’s correction or Fisher’s exact test as appropriate.

Pearson’s or Spearman’s correlations were obtained for the clinical characteristics, echocardiographic parameters and serum Galectin-3 and NT-proBNP levels in the patients with AS.

The cutoff point at which the level of Galectin-3 and NT-proBNP best predicted the occurrence of events in the population was determined using receiver operating characteristic (ROC) curve analysis. The area under the curve and the sensitivity and specificity values were calculated, all with 95% confidence intervals.

A Kaplan–Meier analysis was performed to study the event-free survival curve (time-to-first-event), in this case, the combined endpoint by groups was based on their Galectin-3 levels, NT-proBNP levels and the combination of both, according to the cutoff points obtained from the ROC curve analysis. The differences between the two groups were evaluated with the log-rank test.

A univariate Cox proportional hazards regression analysis was performed to detect the independent predictors of major adverse cardiac events (MACEs). A multivariate model was made with all the significant variables in the univariate analysis. We then removed the variables that did not provide information to the model (*p*-value < 0.22) and created overfitting, as was the case for Vmax and the integral relation, which created significant distortions in the form of outliers.

## 3. Results

The demographic, clinical and geriatric characteristics, laboratory data and echocardiographic findings are detailed in Table 1. The patients in the AS group had significantly more coronary disease, worse functional class, a higher degree of dependence, according to the Barthel index, and greater comorbidity according to the Charlson index. No differences were found in terms of cardiovascular risk factors or the treatment received by the patients. The Galectin-3 and NT-proBNP levels were remarkably higher in the AS group than in the control cohort. The glomerular filtration rates below 50 mL/min were significantly more frequent in the AS group.

The AVA index ranged between 0.23 and 0.65 cm^2^/m^2^. The peak and mean aortic pressure gradients were 57.96 (8.95) mmHg and 33.36 (12.9) mmHg, respectively. As expected, the AS group showed significantly increased LV wall thickness, with a higher percentage of hypertrophy severity. The LVEF was slightly higher in the control group, but not significantly so. The sPAP was higher in the case group. Although the GLS was lower in the AS patients than in the control group, the difference was not significant. When a value of −18% was used as the cutoff point, there was a significant difference between both groups, with the group with AS presenting a higher GLS percentage below this limit (19 vs. 7 patients).

Among the 50 patients with AS, 3 (6%) died during the follow-up year, 4 (8%) were admitted for CHF, 2 (4%) underwent AVR surgery and 10 (20%) underwent TAVI after developing symptoms. The causes of death were not of cardiac origin in any of the three patients.

We further tested the correlations between the Galectin-3 and NT-proBNP levels and significant parameters of AS severity (mean gradients and AVA), as well as the indices of LV remodeling, such as the thickness of the interventricular septum (IVS) and the GLS (Table 2).

There were moderate correlations between the echocardiographic parameters of AS severity and the tested biomarkers, with the NT-proBNP demonstrating greater correlations than the Galectin-3. Both the Galectin-3 and NT-proBNP levels were also positively correlated with IVS, but neither was correlated with the GLS. NT-proBNP but not Galectin-3 was significantly correlated with age.

The univariate analysis by logistic regression yielded a statistically significant direct correlation between NT-proBNP and the occurrence of MACEs, while no such correlation was identified for Galectin-3. Most of the echocardiographic parameters associated with the severity of stenosis (MPG, Vmax, AVA, indexed AVA and the ratio of integrals) were associated with MACEs, while no association was found between impaired GLS and MACEs. In the multivariable analysis, only the level of NT-proBNP remained statistically significant when treated as a continuous variable (Table 3).

**Figure 1:**
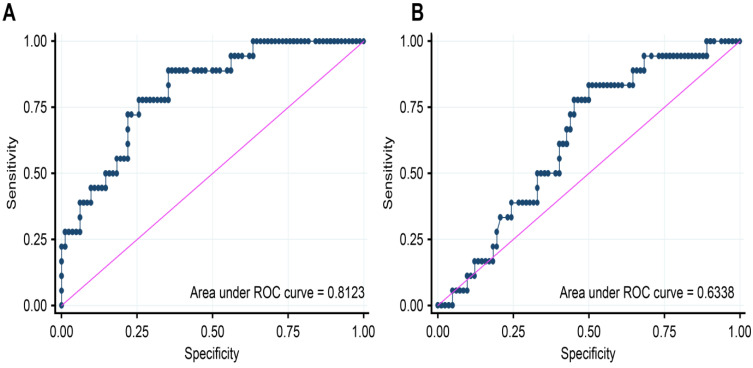
NT-proBNP (**A**) and Galectin-3 (**B**) receiver operating characteristic curve analysis (blue line). Pink line is the reference line. The NT-proBNP and Galectin-3 AUCs were 0.8123 and 0.6338, respectively. Abbreviations: AUC, area under the curve; NT-proBNP, N-terminal prohormone B-type natriuretic peptide; ROC, receiver operating curve.

The area under the receiver operating characteristic curves was 0.81 (95% CI: 0.7111–0.9135) for the NT-proBNP and 0.63 (95% CI: 0.5082–0.759) for the Galectin-3 (Figure 1). The sensitivity, specificity, positive predictive value and negative predictive value of the NT-proBNP and Galectin-3 in predicting events are shown in Table 3. The NT-proBNP was a better event discriminator, with high sensitivity, although the specificity was not very high (Table 4).

**Figure 2:**
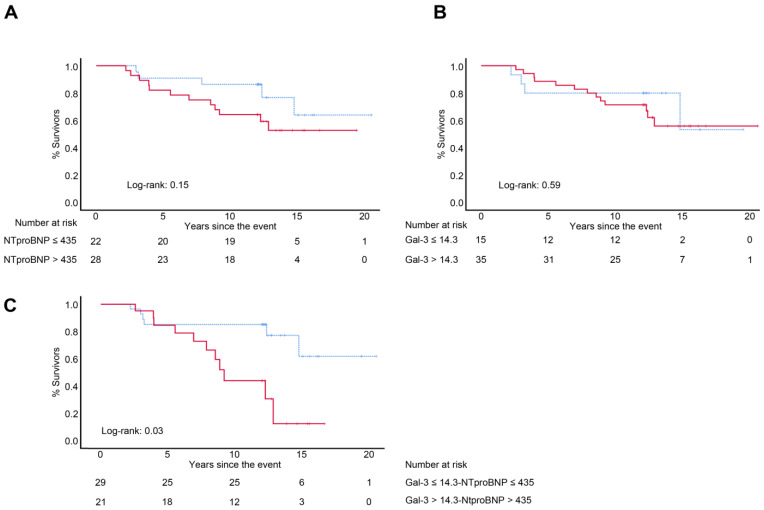
Event-free survival (composite endpoint of AS-related events defined as death from any cause, admission for CHF or appearance of symptoms and change in treatment) according to the cutoff points of NT-proBNP (**A**) and Galectin-3 (**B**) and their combination (**C**). NT-proBNP > 435 (continuous red line) and ≤435 (discontinuous blue line) in patients with asymptomatic severe aortic stenosis. Log-rank *p* = 0.15. Galectin-3 > 14.3 (continuous red line) and ≤14.3 (discontinuous blue line). Log-rank *p* = 0.59. Gal-3 > 14. 3, NT-proBNP > 435 (continuous line) and Gal-3 ≤ 14.3, NT-proBNP ≤ 435 (discontinuous line). Log-rank *p* = 0.032. Abbreviations: AS, aortic stenosis; CHF, congestive heart failure; Gal-3: Galectin-3; NT-proBNP: N-terminal prohormone B-type natriuretic peptide.

Next, to identify the best discriminators of MACEs in our elderly population, the cutoff point that maximized sensitivity and specificity was determined for NT-proBNP (435 pg/mL) and Galectin-3 (14.3 ng/mL). A Kaplan–Meier analysis was used to assess the correlation over time between Galectin-3 ≥ 14.3 ng/mL, NT-proBNP > 435 pg/mL and the combination of both and overall MACE occurrence. Although the NT-proBNP produced a yield curve, no statistically significant difference was found between the groups (*p* = 0.15). The levels of Galectin-3, however, were not predictive of MACEs (*p* = 0.59). Notably, the combination of both biomarkers led to a statistically significant prediction of events (*p* = 0.032) (Figure 2).

## 4. Discussion

The valvular disease AS is a newly emerging pandemic in elderly individuals due to the aging of the population in the Western world. Performing a correct diagnosis in the frail and aged population with AS is a vital problem that often leads to a timing delay in delivering interventional options. Our study assesses the prognostic reliability of the biomarkers NT-proBNP and Galectin-3 in asymptomatic elderly patients with severe AS, which has not been researched to date.

In our study population, we found increased levels of both biomarkers in older patients with asymptomatic AS compared to the control group. Although only NT-proBNP was a good event discriminator in the multivariate analysis, the combination of both biomarkers depicted powerful stratification capabilities using a Galectin-3 level of 14.3 pg/mL and an NT-proBNP level of 435 pg/mL. The role of these two biological markers in increasing diagnostic accuracy has been previously revealed by studies such as that of Feola et al. [24], carried out in patients with CHF.

The diagnosis of severe AS is challenging and requires careful exclusion of measurement errors and other explanations for the echocardiographic findings [17], as well as the presence or absence of typical symptoms which, as we have pointed out, could be difficult to detect in this population. The fact that the mean gradient of our population with AS was below the limit to consider severe stenosis (33.3 ± 12.9 mmHg) was due to the inclusion of AS patients with low gradient and low flow. This entity is very common in elderly patients with hypertension and small and hypertrophic ventricles, predominantly in the female population, as was the case among our patients [25]. This scenario may raise doubts about the diagnosis of severity, so an AVA < 0.8 cm^2^ was required in our study to classify these patients with low gradient and low flow as having severe AS [26]. Clinical decision-making in discordant cases should therefore take additional parameters, such as biomarkers, into account.

Among the studies that systematically reviewed the role of Galectin-3 in AS biological processes, most of the studies reported all-cause mortality [27], but few have analyzed the prediction of events [27]. Galectin-3 levels were significantly higher in patients with AS than in the controls and were correlated moderately with echocardiographic parameters of LV remodeling, such as IVS hypertrophy and severity of stenosis assessed by MPG and AVA, but failed to predict MACEs [28]. Galectin-3 levels are higher in severe AS, highlighting its role as a proinflammatory and profibrotic mediator and confirming the inflammatory process that is underscored in this entity [29].

Galectin-3 has been shown to be related to chronic CHF and myocardial fibrosis in numerous publications, but studies in asymptomatic patients with severe AS are scarce; most of them have been carried out when following up operated patients or patient populations spanning several degrees of AS severity [13,30,31]. As previously mentioned, Galectin-3 is known to be involved in inflammation and the development of fibrosis, but its clinical use is hindered by its lack of specificity in identifying myocardial fibrosis. Several studies have linked the circulating concentration of Galectin-3 to the outcomes in patients with CHF with either reduced or preserved LVEF [31,32]. However, the plasmatic levels of this biomarker may be affected by other factors. Several confounding factors linked to inflammation and fibrosis in other organs, such as female sex, age or renal function, mostly influence Galectin-3 levels [31,32]; impaired renal function is common in patients with cardiovascular disease [33], which may underlie the prognostic value of Galectin-3 in CHF. In our study population, although the presence of eGFR < 50 mL/min was more prevalent among AS patients, the mean global eGFR in this group was within normal limits (57.96 ± 8.9 mL/min). Therefore, in our case, kidney disease did not appear to be a confounding factor.

Galectin-3 levels in the general population described in other studies vary from 10 to 13 ng/mL to 15–30 ng/mL in patients with CHF [34,35,36]. However, the levels in our patients with AS were relatively low, 16.92 ± 4.57, since our patients were asymptomatic when recruited. The lack of association is likely because Galectin-3 increases in more advanced stages of the disease, with LV remodeling and a worsening of renal function secondary to the cardio-renal syndrome.

Arangalage et al. [31] independently studied a subset of 60 asymptomatic patients with severe AS with characteristics similar to those of our populations. Galectin-3 was not associated with either the grade of AS or functional situation and did not provide prognostic information on AS-related events [31]. Bobrowska et al. [32] set out to test the role of Galectin-3 in degenerative AS symptomatic patients as a way to stratify risk for the choice of optimal therapy. Galectin-3 arose as a good predictor of events, specifically of mortality, but only in post-balloon aortic valvuloplasty patients. In a global study group, Galectin-3 tended to predict mortality, which was overturned upon adjustment for eGFR [32].

Despite these unsatisfactory results in predicting events, the known relationship between Galectin-3 and the presence of inflammation and fibrosis could have potential usefulness in leading asymptomatic AS patients with high Galectin-3 levels to undergo cardiac magnetic resonance imaging (MRI) using gadolinium to assess LV fibrosis before TAVI. The presence of myocardial fibrosis is associated with incomplete functional recovery and worse outcomes after TAVI [37]. Routine use of MRI is difficult due to its high cost and low availability, so Galectin-3 could be useful for selecting appropriate candidates. Studies comparing the levels of Galectin-3 before and after TAVI, together with MRI, should be conducted to correlate the degree of fibrosis with this biomarker and the outcomes. Galectin-3 may identify the underlying molecular pathways involved in severe AS and, in the future, be targeted as a therapeutic option.

Unlike Galectin-3, NT-proBNP performed well, yielding more reasonable sensitivity and specificity in predicting the occurrence of MACEs, which is consistent with previous studies [9,38,39]. In the meta-analysis, seventeen studies analyzing NT-proBNP in patients with AS reported an effect size for all-cause mortality in patients with high vs. low levels of baseline biomarkers [27]. Currently, the European clinical practice guidelines recommend at level IIA the use of serial determinations of NT-proBNP to monitor those asymptomatic populations, and, in cases of consistently elevated levels, consider intervention [4]. In our study, we showed that this biomarker was also useful in older patients, despite not having a very high specificity. However, it had a high negative predictive value, and, therefore, levels below the range allowed a “wait and see” attitude during follow-up. The low specificity of NT-proBNP, especially in elderly patients, has led to the use of higher levels in repeated measurements to correlate it with events [4,40]. However, in general, biomarker levels are much lower in valvular disease than in CHF [41]. Thus, the absolute levels of NT-proBNP used for CHF may not be as effective for valvular disease. In our study, the cutoff point that best-discriminated events was 435 pg/mL, similar to the value used for CHF in elderly patients (450 pg/mL), with a sensitivity of 88% and specificity of 66%. NT-proBNP was a better event discriminator in our cohort than in other studies, such as that of Weber et al. [42], which showed an optimized threshold of 550 pg/mL, a sensitivity and specificity of 71% and 68%, respectively, and an AUC of 0.73. In contrast, the low specificity of this biomarker and the modest correlations with AS grading, with coefficients ranging between 0.30 and 0.60, similar to those found in previous studies [42,43,44], suggest that factors other than AS severity may also affect NT-proBNP levels, and thus this biomarker should be used with caution.

Therefore, to make NT-proBNP accessible in clinical practice, it is essential to personalize and consider the whole context, such as age, sex, the presence of AF, renal failure and diastolic dysfunction [43,44]. Several factors could be involved in the NT-proBNP increase, and it is very likely that a set of biomarkers may be needed to achieve high predictive capacity instead of relying on a single biomarker. Previous studies have shown that the combination of different biomarkers improves diagnostic and prognostic performance and may be useful in monitoring patients with heart disease [24,45]. The combination of Galectin-3 with NT-proBNP, therefore, could be introduced into the clinical follow-up of AS patients. Accordingly, Baldenhofer et al. [46] found that a combination of NT-proBNP, mid-regional pro-adrenomedullin and mid-regional pro-atrial natriuretic peptide was a stronger predictor of 1-year mortality than each of these biomarkers separately. No single prognostic marker should be taken as an absolute decision maker, just as no single symptom should be considered to reach a diagnosis in this particular population. Physicians should integrate all possible patient features, including age, comorbidity, frailty, AS severity and impact on the LV, as well as the patient’s desires, into each clinical decision. Our data clearly showed that NT-proBNP levels should be one of the elements that participate in this comprehensive assessment of patients with AS. We propose an integrative medicine approach with multidisciplinary information, such as that from cardiologists, radiologists and laboratory markers.

The current study has some limitations related to the recruitment of the sample from a single center. Additional investigations of NT-proBNP and Galectin-3 levels were only performed at enrollment and were not repeated afterward by the investigators involved in the current study. Further research on a larger population and with an extended period of follow-up is needed to confirm the results of the current study. Patients were considered asymptomatic based on clinical judgment, and no exercises were performed due to the inability of this population to adapt to the treadmill.

## 5. Conclusions

This research confirms the need to establish the optimal cutoff point of circulating biomarkers, as well as to determine its incremental value over other risk stratification methods. In our study, only NT-proBNP was a predictor of events in patients with asymptomatic severe AS. Moreover, a combination of both biomarkers may be a potential tool for the clinical follow-up of the elderly population with AS. Although Galectin-3 was moderately correlated with the echocardiographic parameters, its role as a predictor of MACEs was not confirmed. The proper use of biomarkers in clinical practice could prevent the irreversible and severe consequences that result from late surgical intervention.

## Figures and Tables

**Table 1:** Characteristics of the population.

Characteristics	AS, n (%)	Control, n (%)	*p*
Sex (Female)	32 (64)	32 (64)	1
Age	82.86 ± 7.2	80.48 ± 6.42	0.084
Body surface	1.68 ± 0.19	1.67 ± 0.18	0.82
Functional class			
I	19 (38)	44 (88)	**<0.001**
II	31 (62)	6 (12)	
Coronary heart disease	6 (12)	0 (0)	**0.027**
AF	14 (28)	11 (22)	0.488
DM	18 (36)	11 (22)	0.122
Dyslipidemia	27 (54)	29 (58)	0.68
HBP	40 (80)	38 (76)	0.629
COPD	11 (22)	4 (8)	**0.0499**
CVD	5 (10)	6 (12)	0.74
Dementia	7 (14)	3 (6)	0.31
Degree of dependence			
Independent	28 (56)	42 (84)	**0.0123**
Mild	12 (24)	5 (10)	
Moderate	6 (12)	3 (6)	
Severe	4 (8)	0 (0)	
Barthel index	91.86 ± 16.88	97.86 ± 6.02	0.0199
Comorbidity according to Charlson index			
No comorbidity	27 (54)	39 (78)	
Low comorbidity	14 (28)	8 (16)	**0.033**
High comorbidity	9 (18)	3 (6)	
Charlson index	1.36 ± 1.31	0.84 ± 0.89	**0.022**
Medication			
ACE inhibitors/ARBs	34 (68)	29 (58)	0.3004
Calcium channel blockers	12 (24)	9 (18)	0.461
Potassium-sparing diuretics	1 (2)	3 (6)	0.617
Statins	21 (42)	25 (50)	0.422
Beta-blockers	15 (30)	17 (34)	0.668
Loop diuretics	7 (14)	1 (2)	0.071
Thiazides	14 (28)	14 (28)	0.132
Echocardiography			
LVH			
No	2 (4)	19 (38)	
Mild (11–13 mm)	15 (30)	29 (28)	**<0.001**
Moderate (13–15 mm)	20 (40)	2 (4)	
Severe > 15 mm	13 (26)	0 (0)	
PHT			
Normal	15 (30)	27 (54)	
Mild (35–40)	12 (24)	5 (10)	
Moderate (45–65)	6 (12)	1 (2)	**0.0199**
Severe > 65	1 (2)	0 (0)	
Not estimated	16 (32)	17 (34)	
IVS	13.7 ± 1.66	11.1 ± 1.22	**<0.001**
Peak gradient (mmHg)	57.7 ± 20.32	9.35 ± 3.77	**<0.001**
Mean gradient (mmHg)	33.3 ± 12.9	4.65 ± 1.83	**<0.001**
Peak velocity (m/s)	3.7 ± 0.69	1.48 ± 0.31	**<0.001**
AVA (cm^2^)	0.82 ± 0.26	2.1 ± 0.39	**<0.001**
AVA indexed	0.49 ± 0.16	1.3 ± 0.28	**<0.001**
Integral relation	0.25 ± 0.08	0.66 ± 0.13	**<0.001**
LVEF Teicholz (%)	67.9 ± 9.8	71.2 ± 7.64	0.07
Indexed stroke volume	39.5 ± 10.68	39.4 ± 9.31	0.966
GLS	18.67 ± 3.25	19.48 ± 1.78	0.136
GLS < 18%	19 (38.8)	7 (14)	**0.0051**
Lab results			
GFR (mL/min)	57.96 ± 8.9	59.6 ± 8.49	0.53
GFR < 50 mL/min	15 (30)	7 (14)	**0.045**
NTproBNP	1117.28 ± 1528.49	271.9 ± 350.8	**<0.001**
NTproBNP > 450	28 (56)	6 (12)	**<0.001**
NTproBNP > 435	26 (52)	5 (10)	**<0.001**
Galectin-3			
Mild risk ≤ 17.8	27 (54)	43 (86)	**0.0011**
Moderate risk > 17.8–25.9	21 (42)	7 (14)	
High risk > 25.9	2 (4)	0	
Galectin-3	16.91 ± 4.57	12.7 ± 4.74	**<0.001**
Galectin-3 > 14.3	35 (70)	15 (30)	**<0.001**
ECG			
Heart rate	69.8 ± 12.13	69.22 ± 12.04	0.81
Rhythm			
Sinusal	42 (84)	46 (92)	
AF	6 (12)	4 (8)	0.275
Pacemaker	2 (4)	0 (0)	
LVH criteria	7 (14)	2 (4)	0.16

Abbreviations: ACE inhibitors/ARBs, angiotensin-converting enzyme (ACE) inhibitors/angiotensin II receptor blocker; AF, atrial fibrillation; AS, aortic stenosis; AVA, aortic valvular area; COPD, chronic obstructive pulmonary disease; CVD, cerebrovascular disease; DM, diabetes mellitus; ECG, electrocardiogram; GFR, glomerular filtration rate; GLS, global longitudinal strain; HBP, high blood pressure; IVS, interventricular septum; LVEF, left ventricular ejection fraction; LVH, left ventricular hypertrophy; NT-proBNP, N-terminal prohormone B-type natriuretic peptide; PHT, pulmonary hypertension. Notes: Data are expressed as No. (%) or mean ± standard deviation. Bold indicates statistically significant variables (*p* < 0.05).

**Table 2:** Correlations between AS patient features, echocardiography parameters and plasma levels of Galectin-3 and NT-proBNP.

		Age	Mean Gradient	AVA	GLS	IVS
NT-proBNP	S	0.419	0.455	−0.474	−0.181	0.574
	*p*	<0.0001	<0.0001	<0.0001	0.07	<0.0001
Galectin-3	S	0.183	0.339	−0.366	−0.029	0.369
	*p*	0.069	0.0006	0.0002	0.77	0.0002

Abbreviations: AS, aortic stenosis; AVA, aortic valve area; GLS, global longitudinal strain; IVS, interventricular septum; NT-proBNP, N-terminal prohormone B-type natriuretic peptide. Data are shown as Pearson’s or Spearman’s correlation coefficients (r) for parametric and nonparametric characteristics, respectively, and associated *p* values.

**Table 3:** Independent predictors of MACEs (univariate and multivariate analysis with Cox regression).

Features	Univariate		Multivariate	
	HR	*p*-Value	HR	*p*-Value
Maximum pressure gradient	1.03 (1.00; 1.05)	0.0277		
MPG	1.06 (1.02; 1.10)	0.0045		
Vmax	2.48 (1.17; 5.27)	0.0184		
AVA	0.02 (0.00; 0.23)	0.0018		
AVA indexed	0.001 (0.000; 0.088)	0.0019		
Integral relation	0.000 (0.000; 0.002)	0.0003		
LVEF Teichholz	0.94 (0.89; 1.00)	0.0329		
Indexed stroke volume	0.95 (0.90; 0.99)	0.0242		
GLS	0.90 (0.77; 1.05)	0.1882		
NT-proBNP	1.000 (1.000; 1.000)	0.0125	3.45 (1.32; 9.03)	0.011
Galectin-3	0.98 (0.89; 1.09)	0.7679	0.97 (0.87; 1.08)	0.609

Abbreviations: AVA, aortic valvular area; GLS, global longitudinal strain; HR, hazard ratio; LVEF, left ventricular ejection fraction; MACE, major cardiovascular adverse events; MPG, mean pressure gradient; NT-ProBNP, N-terminal B-type natriuretic peptide; Vmax, maximum velocity.

**Table 4:** The predictive ability of the biochemistry parameters for the outcomes of the asymptomatic severe AS cohort.

	ROC AREA	Youden Index	Sensitivity	Specificity	PPV	NPV	Cut Point AUC
NT-proBNP	0.8123	0.5352	88.8889	64.6341	35.5556	96.3636	435.00
Galectin-3	0.6338	0.3333	83.3333	50.0000	26.7857	93.1818	14.30

Abbreviations: AS, aortic stenosis; AUC, area under the curve; NPV, negative predictive value; NT-proBNP, N-terminal prohormone B-type natriuretic peptide; PPV, positive predictive value; ROC, receiver operating characteristic.

## Data Availability

The data that support the findings of this study are available from the corresponding author upon request. The data are not publicly available due to privacy or ethical restrictions.

## References

[1] Iung B., Baron G., Butchart E.G., Delahaye F., Gohlke-Bärwolf C., Levang O.W., Tornos P., Vanoverschelde J.L., Vermeer F., Boersma E. (2003). A prospective survey of patients with valvular heart disease in Europe: The Euro Heart survey on valvular heart disease. Eur. Heart J..

[2] Pellikka P.A., Sarano M.E., Nishimura R.A., Malouf J.F., Bailey K.R., Scott C.G., Barnes M.E., Tajik A.J. (2005). Outcome of 622 adults with asymptomatic, hemodynamically significant aortic stenosis during prolonged follow-up. Circulation.

[3] Rosenhek R., Zilberszac R., Schemper M., Czerny M., Mundigler G., Graf S., Bergler-Klein J., Grimm M., Gabriel H., Maurer G. (2010). Natural history of very severe aortic stenosis. Circulation.

[4] Vahanian A., Beyersdorf F., Praz F., Milojevic M., Baldus S., Bauersachs J., Capodanno D., Conradi L., De Bonis M., De Paulis R. (2022). 2021 ESC/EACTS guidelines for the management of valvular heart disease. Eur. Heart J..

[5] Leyhe T., Reynolds C.F., Melcher T., Linnemann C., Klöppel S., Blennow K., Zetterberg H., Dubois B., Lista S., Hampel H. (2017). A common challenge in older adults: Classification, overlap, and therapy of depression and dementia. Alzheimer’s Dement. J. Alzheimer’s Assoc..

[6] Aiello G., Cuocina M., La Via L., Messina S., Attaguile G.A., Cantarella G., Sanfilippo F., Bernardini R. (2023). Melatonin or Ramelteon for Delirium Prevention in the Intensive Care Unit: A Systematic Review and Meta-Analysis of Randomized Controlled Trials. J. Clin. Med..

[7] Ramos M., Quezada D.M., Ayala R., Gómez-Pavón F.J., Jaramillo J., Toro R. (2019). Aortic stenosis prognosis in older patients: Frailty is a strong marker of early congestive heart failure admissions. Eur. Geriatr. Med..

[8] Lim P., Monin J., Monchi M., Garot J., Pasquet A., Hittinger L., Vanoverschelde J., Carayon A., Gueret P. (2004). Predictors of outcome in patients with severe aortic stenosis and normal left ventricular function: Role of B-type natriuretic peptide. Eur. Heart J..

[9] Lancellotti P., Moonen M., Magne J., O’Connor K., Cosyns B., Attena E., Donal E., Pierard L. (2010). Prognostic effect of long-axis left ventricular dysfunction and B-type natriuretic peptide levels in asymptomatic aortic stenosis. Am. J. Cardiol..

[10] Cimadevilla C., Cueff C., Hekimian G., Dehoux M., Lepage L., Iung B., Duval X., Huart V., Tubach F., Vahanian A. (2013). Prognostic value of B-type natriuretic peptide in elderly patients with aortic valve stenosis: The COFRASA–GENERAC study. Heart.

[11] Dong R., Zhang M., Hu Q., Zheng S., Soh A., Zheng Y., Yuan H. (2018). Galectin-3 as a novel biomarker for disease diagnosis and a target for therapy (Review). Int. J. Mol. Med..

[12] Lopez-Andrès N., Rossignol P., Iraqi W., Fay R., Nuée J., Ghio S., Cleland J.G.F., Zannad F., Lacolley P. (2012). Association of galectin-3 and fibrosis markers with long-term cardiovascular outcomes in patients with heart failure, left ventricular dysfunction, and dyssynchrony: Insights from the CARE-HF (Cardiac Resynchronization in Heart Failure) trial. Eur. J. Heart Fail..

[13] Sádaba J.R., Martínez-Martínez E., Arrieta V., Álvarez V., Fernández-Celis A., Ibarrola J., Melero A., Rossignol P., Cachofeiro V., López-Andrés N. (2016). Role for galectin-3 in calcific aortic valve stenosis. J. Am. Heart Assoc..

[14] Filipe M.D., Meijers W.C., Rogier van der Velde A., de Boer R.A. (2015). Galectin-3 and heart failure: Prognosis, prediction & clinical utility. Clin. Chim. Acta.

[15] Charlson M.E., Pompei P., Ales K.L., MacKenzie C.R. (1987). A new method of classifying prognostic comorbidity in longitudinal studies: Development and validation. J. Chronic Dis..

[16] Mahoney F., Barthel D. (1965). Functional evaluation: The barthel index. Md. State Med. J..

[17] Baumgartner H., Hung J., Bermejo J., Chambers J.B., Edvardsen T., Goldstein S., Lancellotti P., LeFevre M., Miller F., Otto C.M. (2017). Recommendations on the echocardiographic assessment of aortic valve stenosis: A focused update from the European association of cardiovascular imaging and the American society of echocardiography. Eur. Heart J. Cardiovasc. Imaging.

[18] Lang R.M., Badano L.P., Mor-Avi V., Afilalo J., Armstrong A., Ernande L., Flachskampf F.A., Foster E., Goldstein S.A., Kuznetsova T. (2015). Recommendations for cardiac chamber quantification by echocardiography in adults: An update from the American society of echocardiography and the European association of cardiovascular imaging. J. Am. Soc. Echocardiogr..

[19] Nagueh S.F., Smiseth O.A., Appleton C.P., Byrd B.F., Dokainish H., Edvardsen T., Flachskampf F.A., Gillebert T.C., Klein A.L., Lancellotti P. (2016). Recommendations for the evaluation of left ventricular diastolic function by echocardiography: An update from the American society of echocardiography and the European association of cardiovascular imaging. J. Am. Soc. Echocardiogr..

[20] Voigt J.U., Pedrizzetti G., Lysyansky P., Marwick T.H., Houle H., Baumann R., Pedri S., Ito Y., Abe Y., Metz S. (2015). Definitions for a common standard for 2D speckle tracking echocardiography: Consensus document of the EACVI/ASE/Industry Task Force to standardize deformation imaging. Eur. Heart J. Cardiovasc. Imaging.

[21] Galiè N., Humbert M., Vachiery J.L., Gibbs S., Lang I., Torbicki A., Simonneau G., Peacock A., Vonk Noordegraaf A., Beghetti M. (2016). 2015 ESC/ERS Guidelines for the diagnosis and treatment of pulmonary hypertension: The joint task force for the diagnosis and treatment of pulmonary hypertension of the European Society of Cardiology (ESC) and the European Respiratory Society (ERS): Endorsed by: Association for European Paediatric and Congenital Cardiology (AEPC), International Society for Heart and Lung Transplantation (ISHLT). Eur. Heart J..

[22] McCullough P.A., Olobatoke A., Vanhecke T.E. (2011). Galectin-3: A novel blood test for the evaluation and management of patients with heart failure. Rev. Cardiovasc. Med..

[23] Levey A.S., Stevens L.A., Schmid C.H., Zhang Y.L., Castro A.F., Feldman H.I., Kusek J.W., Eggers P., Van Lente F., Greene T. (2009). A new equation to estimate glomerular filtration rate. Ann. Intern. Med..

[24] Feola M., Testa M., Leto L., Cardone M., Sola M., Rosso G.L. (2016). Role of galectin-3 and plasma B type-natriuretic peptide in predicting prognosis in discharged chronic heart failure patients. Medicine.

[25] Clavel M.A., Dumesnil J.G., Capoulade R., Mathieu P., Sénéchal M., Pibarot P. (2012). Outcome of patients with aortic stenosis, small valve area, and low-flow, low-gradient despite preserved left ventricular ejection fraction. J. Am. Coll. Cardiol..

[26] Minners J., Allgeier M., Gohlke-Baerwolf C., Kienzle R.P., Neumann F.J., Jander N. (2010). Inconsistent grading of aortic valve stenosis by current guidelines: Haemodynamic studies in patients with apparently normal left ventricular function. Heart.

[27] White M., Baral R., Ryding A., Tsampasian V., Ravindrarajah T., Garg P., Koskinas K.C., Clark A., Vassiliou V.S. (2021). Biomarkers associated with mortality in aortic stenosis: A systematic review and meta-analysis. Med. Sci..

[28] Agoston-Coldea L., Bheecarry K., Petra C., Strambu L., Ober C., Revnic R., Lupu S., Mocan T., Fodor D. (2018). The value of global longitudinal strain and galectin-3 for predicting cardiovascular events in patients with severe aortic stenosis. Med. Ultrason..

[29] Toro R., Mangas A., Gómez F. (2011). Enfermedad de la válvula aórtica calcificada. Su asociación con la arteriosclerosis. Med. Clín..

[30] Martínez-Martínez E., López-Ándres N., Jurado-López R., Rousseau E., Bartolomé M.V., Fernández-Celis A., Rossignol P., Islas F., Antequera A., Prieto S. (2015). Galectin-3 participates in cardiovascular remodeling associated with obesity. Hypertension.

[31] Arangalage D., Nguyen V., Robert T., Melissopoulou M., Mathieu T., Estellat C., Codogno I., Huart V., Duval X., Cimadevilla C. (2016). Determinants and prognostic value of Galectin-3 in patients with aortic valve stenosis. Heart.

[32] Bobrowska B., Wieczorek-Surdacka E., Kruszelnicka O., Chyrchel B., Surdacki A., Dudek D. (2017). Clinical correlates and prognostic value of plasma galectin-3 levels in degenerative aortic stenosis: A single-center prospective study of patients referred for invasive treatment. Int. J. Mol. Sci..

[33] Heywood J.T., Fonarow G.C., Costanzo M.R., Mathur V.S., Wigneswaran J.R., Wynne J. (2007). High prevalence of renal dysfunction and its impact on outcome in 118,465 patients hospitalized with acute decompensated heart failure: A report from the ADHERE database. J. Card. Fail..

[34] de Boer R.A., Lok D.J.A., Jaarsma T., van der Meer P., Voors A.A., Hillege H.L., van Veldhuisen D.J. (2011). Predictive value of plasma galectin-3 levels in heart failure with reduced and preserved ejection fraction. Ann. Med..

[35] de Boer R.A., van Veldhuisen D.J., Gansevoort R.T., Muller Kobold A.C., van Gilst W.H., Hillege H.L., Bakker S.J.L., van der Harst P. (2012). The fibrosis marker galectin-3 and outcome in the general population. J. Intern. Med..

[36] Ho J.E., Liu C., Lyass A., Courchesne P., Pencina M.J., Vasan R.S., Larson M.G., Levy D. (2012). Galectin-3, a marker of cardiac fibrosis, predicts incident heart failure in the community. J. Am. Coll. Cardiol..

[37] Barone-Rochette G., Piérard S., De Meester de Ravenstein C., Seldrum S., Melchior J., Maes F., Pouleur A.-C., Vancraeynest D., Pasquet A., Vanoverschelde J.-L. (2014). Prognostic significance of LGE by CMR in aortic stenosis patients undergoing valve replacement. J. Am. Coll. Cardiol..

[38] Clavel M.-A., Malouf J., Michelena H.I., Suri R.M., Jaffe A.S., Mahoney D.W., Enriquez-Sarano M. (2014). B-type natriuretic peptide clinical activation in aortic stenosis. J. Am. Coll. Cardiol..

[39] Henri C., Dulgheru R., Magne J., Caballero L., Laaraibi S., Davin L., Kou S., Voilliot D., Nchimi A., Oury C. (2016). Impact of serial B-type natriuretic peptide changes for predicting outcome in asymptomatic patients with aortic stenosis. Can. J. Cardiol..

[40] Redfield M.M., Rodeheffer R.J., Jacobsen S.J., Mahoney D.W., Bailey K.R., Burnett J.C. (2002). Plasma brain natriuretic peptide concentration: Impact of age and gender. J. Am. Coll. Cardiol..

[41] Detaint D., Messika-Zeitoun D., Chen H.H., Rossi A., Avierinos J.-F., Scott C., Burnett J.C., Enriquez-Sarano M. (2006). Association of B-type natriuretic peptide activation to left ventricular end-systolic remodeling in organic and functional mitral regurgitation. Am. J. Cardiol..

[42] Weber M., Arnold R., Rau M., Brandt R., Berkovitsch A., Mitrovic V., Hamm C. (2004). Relation of N-terminal pro–B-type natriuretic peptide to severity of valvular aortic stenosis. Am. J. Cardiol..

[43] Maréchaux S., Hattabi M., Juthier F., Neicu D.V., Richardson M., Carpentier E., Bouabdallaoui N., Delelis F., Banfi C., Breyne J. (2011). Clinical and echocardiographic correlates of plasma B-type natriuretic peptide levels in patients with aortic valve stenosis and normal left ventricular ejection fraction. Echocardiography.

[44] Wang T.J., Larson M.G., Levy D., Leip E.P., Benjamin E.J., Wilson P.W.F., Sutherland P., Omland T., Vasan R.S. (2002). Impact of age and sex on plasma natriuretic peptide levels in healthy adults. Am. J. Cardiol..

[45] Baran J., Niewiara Ł., Podolec J., Siedliński M., Józefczuk E., Bernacik A., Badacz R., Przewłocki T., Pieniążek P., Żmudka K. (2022). Serum and vascular stiffness biomarkers associated with the severity of degenerative aortic valve stenosis and cardiovascular outcomes. J. Cardiovasc. Dev. Dis..

[46] Baldenhofer G., Laule M., Mockel M., Sanad W., Knebel F., Dreger H., Leonhardt F., Sander M., Grubitzsch H., Baumann G. (2016). Mid-regional pro-adrenomedullin (MR-proADM) and mid-regional pro-atrial natriuretic peptide (MR-proANP) in severe aortic valve stenosis: Association with outcome after transcatheter aortic valve implantation (TAVI). Clin. Chem. Lab. Med..

